# Psilocybin decreases neural responsiveness and increases functional connectivity while preserving pure-tone frequency selectivity in mouse auditory cortex

**DOI:** 10.1152/jn.00124.2024

**Published:** 2024-05-29

**Authors:** Adam T. Brockett, Nikolas A. Francis

**Affiliations:** ^1^Department of Psychology, University of Maryland, College Park, Maryland, United States; ^2^Program in Neuroscience and Cognitive Science, University of Maryland, College Park, Maryland, United States; ^3^Department of Biological Sciences, University of New Hampshire, Durham, New Hampshire, United States; ^4^Department of Biology, University of Maryland, College Park, Maryland, United States; ^5^Center for Comparative and Evolutionary Biology of Hearing, University of Maryland, College Park, Maryland, United States; ^6^Brain and Behavior Institute, University of Maryland, College Park, Maryland, United States

**Keywords:** auditory cortex, behavior, brain, psilocybin, psychedelics

## Abstract

Psilocybin is a serotonergic psychedelic believed to have therapeutic potential for neuropsychiatric conditions. Despite well-documented prevalence of perceptual alterations, hallucinations, and synesthesia associated with psychedelic experiences, little is known about how psilocybin affects sensory cortex or alters the activity of neurons in awake animals. To investigate, we conducted two-photon imaging experiments in auditory cortex of awake mice and collected video of free-roaming mouse behavior, both at baseline and during psilocybin treatment. In comparison with pre-dose neural activity, a 2 mg/kg ip dose of psilocybin initially increased the amplitude of neural responses to sound. Thirty minutes post-dose, behavioral activity and neural response amplitudes decreased, yet functional connectivity increased. In contrast, control mice given intraperitoneal saline injections showed no significant changes in either neural or behavioral activity across conditions. Notably, neuronal stimulus selectivity remained stable during psilocybin treatment, for both tonotopic cortical maps and single-cell pure-tone frequency tuning curves. Our results mirror similar findings regarding the effects of serotonergic psychedelics in visual cortex and suggest that psilocybin modulates the balance of intrinsic versus stimulus-driven influences on neural activity in auditory cortex.

**NEW & NOTEWORTHY** Recent studies have shown promising therapeutic potential for psychedelics in treating neuropsychiatric conditions. Musical experience during psilocybin-assisted therapy is predictive of treatment outcome, yet little is known about how psilocybin affects auditory processing. Here, we conducted two-photon imaging experiments in auditory cortex of awake mice that received a dose of psilocybin. Our results suggest that psilocybin modulates the roles of intrinsic neural activity versus stimulus-driven influences on auditory perception.

## Graphical abstract

**Figure:**
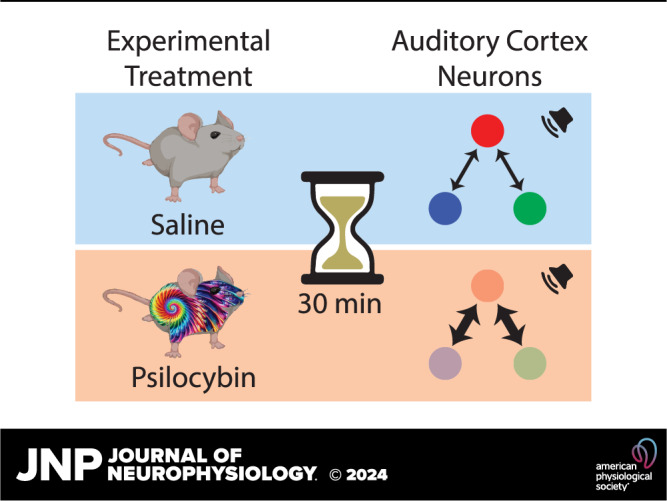

## INTRODUCTION

Psilocybin is a psychoactive prodrug that is well-known for inducing atypical changes in conscious experience, including altered perception, cognition, and mood (1–10). Despite its therapeutic potential (1–13), few studies have investigated the neural basis by which psilocybin produces these profound changes in the brain of awake animals. A recent study in awake mice found that psilocybin increased neuronal spiking in anterior cingulate cortex (ACC) while modulating neural synchrony (14). Functional magnetic resonance imaging (fMRI) studies in awake humans have shown that psilocybin alters brain-wide activity and synchronization (10, 15), and that altered perceptions associated with psilocybin can be suppressed by a block of the 5-HT2a receptor (13, 16). Notably, musical experience during psilocybin-assisted therapy is predictive of treatment outcome (17). Auditory cortex plays a critical role in our perception and recognition of music and sound in general. However, it remains unclear how psilocybin’s effects on neural activity in auditory cortex contributes to therapeutic treatment, or the perceptual alterations, pseudo-hallucinations, and synesthesia that may occur during psychedelic experiences (11, 12, 16, 18).

Here, we used two-photon (2P) Ca^2+^ imaging (19, 20) of neural activity in awake mice to study how psilocybin affects sensory processing in primary auditory cortex (A1) layer 2/3 (L2/3). We also used video analysis of free-roaming mice to quantify the time-course of behavioral effects during psilocybin treatment. We show that psilocybin modulates both cortical and behavioral states, while preserving neural selectivity for auditory stimuli.

## MATERIALS AND METHODS

### Animals

All procedures were approved by the University of Maryland Institutional Animal Care and Use Committee. Psilocybin was procured from the National institute on Drug Abuse drug supply program. We used *n* = 19 mice, which were F1 offspring of CBA/CaJ mice (The Jackson Laboratory; Stock No. 000654) crossed with transgenic C57BL/6J-Tg (Thy1-GCaMP6s)GP4.3Dkim/J mice (19–21) (The Jackson Laboratory; Stock No. 024275), 1.5–7 mo old. Note that this is a GCaMP6 mouse line that does not show aberrant cortical activity (22). We used the F1 generation of the crossed mice because they have healthy hearing at least 1 yr into adulthood (21). Mice were housed under a reversed 12-h light/12-h dark light cycle.

### Video Analysis of Mouse Behavior

We studied mouse behavior using a custom-built video tracking arena. The arena consisted of a large black acrylic box with an open top, that contained a smaller clear acrylic box in the center of the arena. White LED arrays were attached to inside walls of the black box to illuminate the small clear box. Bedding was placed inside the clear box, and a pco.Panda 4.2 CMOS camera was mounted above, with only the extent of the clear box kept in-frame. Videos were taken at 150 frames per second (fps) and analyzed at 30 fps (see Supplemental Videos S1 and S2). We quantified mouse movement in each video, *V*, by first creating a new video, *V*′, from sequential frame differences in *V*, i.e., *V*′(*f*) = *V*(*f* + 1) − *V*(*f*), where *f* indexes each frame. For the data in Fig. 3*D*, we then created a one-dimensional frame difference trace, *D*, by summing over all pixels for each frame in *V*′. We extracted the envelope of *D*, using its low-passed Hilbert transform magnitude, to produce the mouse’s movement trace, *M*, spanning each 40–60 min video. For the spatial movement analysis in Fig. 3*E*, to denoise movement information within the videos, pixels with values less than 2 standard errors of the mean movement across a given video were set to 0 before averaging across videos.

Behavioral experiments began with habituation, by placing the mouse in the small clear box for an hour, before being administered an intraperitoneal (ip) injection of either psilocybin (2 mg/kg in 0.1 mL saline) (*n* = 5 mice) or a 0.1 mL saline vehicle (*n* = 5 mice). Immediately after injection, the mouse was placed back in the clear box, covered with a clear lid, and then videoed for an additional 40–60 min.

### Stimulus Design and Presentation

During imaging experiments, we presented awake mice with 70 dB SPL pure-tones from an ES1 free-field speaker (Tucker-Davis Technologies) connected to an ED1 amplifier (Tucker-Davis Technologies). The speaker was calibrated to give a flat frequency response in the 1–80 kHz range. Stimuli were synthesized in Matlab software (MathWorks) and output from a National instruments board (NI-6211, 200 kHz sampling rate) to the ED1 amplifier. Each pure-tone was 500 ms in duration, with 5 ms and 495 ms raised-cosine attack and decay ramps, respectively. The frequency of each pure-tone was randomly selected from 10 equiprobable values (2–45 kHz, 2 tones per octave). Each frequency was repeated 20 times per experiment, with interstimulus intervals randomized at 6, 7, or 8 s. The full stimulus set was presented within ∼20 min.

### Chronic Window Implantation

Mice were given an intraperitoneal injection of dexamethasone (5 mg/kg) at least 1 h prior to surgery to prevent inflammation and edema. Mice were deeply anesthetized using isoflurane (5% induction, 0.5–2% for maintenance) and given a subcutaneous injection of cefazolin (500 mg/kg). Internal body temperature was maintained at 37.5°C using a feedback-controlled heating blanket. Scalp fur was trimmed using scissors and any remaining fur was removed using Nair. The scalp was disinfected with alternating swabs of 70% ethanol and betadine. A patch of skin over the temporal bone was removed and the underlying bone cleared of connective tissue using a scalpel. The temporal muscle was detached from the skull, and the skull was cleaned and dried. A thin layer of cyanoacrylate glue (VetBond) was applied to the exposed skull surface and a three-dimensional (3-D) printed stainless steel head-plate was affixed to the midline of the skull. Dental cement (C&B Metabond) was used to cover the entire head-plate. A circular craniotomy (3 mm diameter) was made over the auditory cortex where the chronic imaging window was implanted. The window was either of a stack of two 3-mm diameter coverslips or a 3.2-mm diameter, 1-mm thick uncoated sapphire window (Edmund Optics), glued with optical adhesive (Norland 61) to a 5-mm diameter coverslip. The space between the glass and the skull was sealed with a silicone elastomer (Kwik-Sil). The edges of the glass and the skull were then sealed with dental cement. Finally, the entire implant except for the imaging window was coated with black dental cement created by mixing methyl methacrylate with iron oxide powder to reduce optical reflections. Meloxicam (0.5 mg/kg) was given subcutaneously as a postoperative analgesic.

### Widefield Imaging

To localize primary auditory cortex (A1) via rostro-caudal tonotopic gradients, we performed widefield imaging experiments. Awake mice were placed into a 3-D-printed plastic tube and head-restraint system. Blue excitation light was shone by an LED (470 nm) through an excitation filter (470 nm) and directed into the cranial window. Emitted fluorescence (*F*) from neurons in Thy1-GCaMP6s mice was collected through a ×4 objective (Thorlabs), passed through a longpass filter (cutoff: 505 nm), followed by a bandpass emission filter (531 nm) attached to a CMOS camera. Images were acquired using ThorCam software (Thorlabs).

After acquiring an image of the cortical surface, the focal plane was advanced to approximately 750 μm below the surface for imaging neural activity. To visualize tonotopy, pure-tones were presented from a free field speaker, as described earlier. Widefield images were acquired at a 5 Hz rate and 512 × 512 pixels. Using Matlab software, image sequences for each tone frequency were averaged and background illumination was reduced using a homomorphic contrast filter. For each pixel, Δ*F*/*F* traces were calculated by finding the average *F* taken from the silent baseline period before a pure-tone presentation, subtracting that value from subsequent time-points until 3 s after the pure-tone, then dividing all time-points by the baseline *F*. To visualize auditory responses, we kept traces with Δ*F*/*F* within 90% of the maximum response in the pixel-wise grand-average of Δ*F*/*F* (i.e., Δ*F*/*F*_90_). Pixel-wise tonotopic frequencies were taken as the median frequency of the set of tones corresponding to the Δ*F*/*F*_90_ traces.

### Two-Photon Imaging

**Figure 1.**
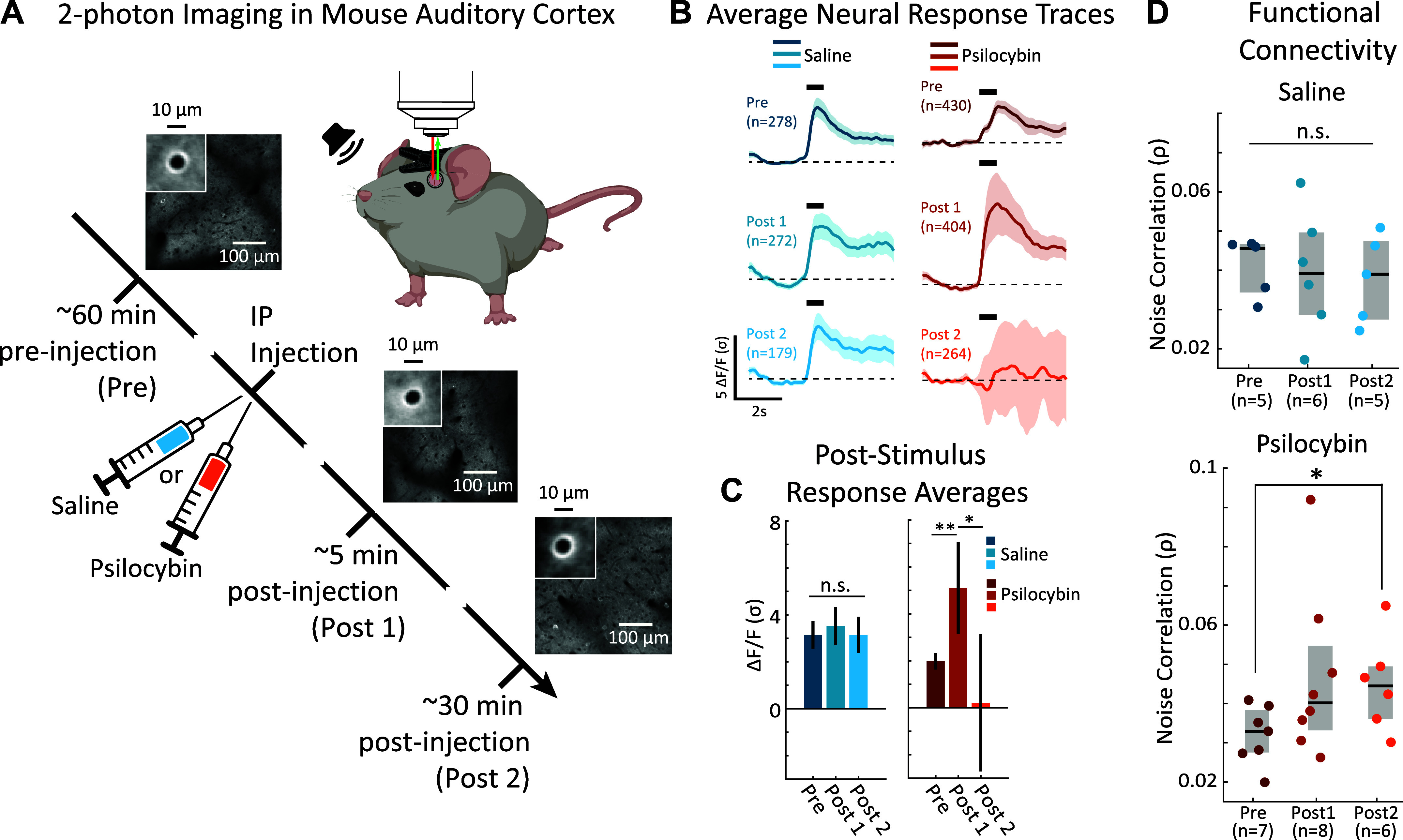
Psilocybin modulates functional connectivity and neural responses to sound in primary auditory cortex (A1) layer 2/3 (L2/3) of awake mice. *A*: experimental paradigm. Mice were 2-photon (2P) imaged preinjection (Pre), then given an intraperitoneal (ip) injection with either 2 mg/kg psilocybin or 0.1 mL saline, then two postinjection imaging sessions occurred (Post 1 and Post 2, respectively). Each micrograph shows an example 2P field of view (FOV) from our experiments. The *inset* cell images in each panel show the average neuron in the FOV. *B*: population-averaged auditory responses traces in A1 L2/3. Shading shows 2 standard errors of the mean (SEM). *C*: poststimulus time-averaged neural population responses. Neuronal fluorescence during the 1 s following stimulus presentation was averaged for each neuron. The mean population response for each condition is shown with 2 SEMs. The stars indicate significant differences in the Psilocybin condition (Pre vs. Post 1: **, bootstrap *t* test *P* = 0.015; Post 2 vs. Post 1: *, bootstrap *t* test *P* = 0.03. Sample sizes are the same as in *B*. *D*: functional connectivity in A1 L2/3. Each panel shows a dot which is the population average noise correlation in each experiment. The black horizontal bar and the gray rectangles show the median and interquartile range, respectively. The star in the lower panel indicates a significant Pre vs. Post 2 difference in noise correlations (bootstrap *t* test, *P* = 0.044). “n.s.” in the *top* indicates no significant differences. Created with BioRender and published with permission.

Recording sites were selected for two-photon (2P) imaging in A1 layer 2/3 (L2/3) for each Thy1-GCaMP6s × CBA mouse. Thy1-GCaMP6s transgenic mice enable imaging of Ca^2+^ fluorescence, primarily in pyramidal neurons and in layers 2/3, 5, and 6 (20). Figure 1*A* illustrates the timeline of our imaging experiments. Awake mice were placed into a 3-D-printed plastic tube and head-restraint system. For each mouse, we began with a preinjection imaging session (Pre). We then waited an hour before injecting either 2 mg/kg psilocybin (*n* = 6 mice, 2 females, 4 males) or the 0.1 mL saline vehicle alone (*n* = 3 mice, 3 males). The injected mouse was then immediately placed back under the microscope for postinjection imaging (Post 1), which began 5–8 min after the injection. With the injected mouse still under the microscope, we then performed a final imaging session (Post 2), which began 34–43 min after injection. Two of six psilocybin treated mice received a dose on two separate experiments done 8 days apart, well beyond the ∼30 min half-life of psilocybin/psilocin in mice (23, 24). Each saline control mouse was imaged twice within several days. Our 2P recording sites were chosen at a different region in A1 for each Pre, Post 1, and Post 2 session.

Our experiments used a scanning microscope (Bergamo II series, Thorlabs) coupled to a pulsed femtosecond 2-photon laser with dispersion precompensation (Coherent Chameleon Discovery NX TPC). The microscope was controlled by ThorImage software. The laser was tuned to λ = 940 nm to excite GCaMP6s. Fluorescence signals were collected through a ×16 0.8 numerical aperture (NA) microscope objective (Nikon). Emitted photons were directed through a 525 nm (green) bandpass filter onto a GaAsP photomultiplier tube. The field of view was 411 × 411 µm. Imaging frames of 512 × 512 pixels (0.8 µm/pixel) were acquired at 30 Hz by bidirectional scanning of an 8 kHz resonant scanner. Laser power was set to ∼70 mW, measured at the objective. During experiments, the objective’s focal plane was lowered into L2/3 (∼100 µm below the surface) before imaging neuronal responses to pure-tones.

After 2P experiments, all images were processed using Matlab. Image motion was corrected using the TurboReg plug-in for MIJI (i.e., FIJI for Matlab). The example 2P fields of view (FOVs) in Fig. 1*A* show the averages of registered images for GCaMP6s images across a session. The inset images show the average neuron in the 2P FOV. The well-defined fluorescent cell membrane indicates clear GCaMP6s expression and accurate cell image segmentation. To extract neuronal fluorescence traces, the centers of cell bodies were manually selected, a then a ring-like region of interest (ROI) was cropped around the cell center. Overlapping ROI pixels (due to neighboring neurons) were excluded from analysis. For each labeled neuron, a raw fluorescence signal over time was extracted from somatic ROIs. Pixels within the ROI were averaged to create individual neuron fluorescence traces, C(*t*), for each trial of the experiment. Neuropil fluorescence was estimated for each cellular ROI using an additional ring-shaped ROI, which began 3 pixels from the somatic ROI. Pixels from the new ROI were averaged to obtain neuropil fluorescence traces, N(*t*), for the same time-period as the individual neuron fluorescence traces. Pixels from regions with overlapping neuropil and cellular ROIs were removed from neuropil ROIs. Neuropil-corrected cellular fluorescence was calculated as C(*t*) = C(*t*) – 0.7 N(*t*) (19, 20, 25). Only cells with positive values obtained from averaging C(*t*) across time were kept for analysis, since negative values may indicate neuropil contamination. Δ*F*/*F* was calculated from C(*t*), for each neuron, by finding the average *F* taken from the silent baseline period before a pure-tone presentation, subtracting that value from subsequent time-points until 3 s after the pure-tone, then dividing all time-points by the baseline *F*. Δ*F*/*F* was normalized by the standard deviation of response amplitude (σ) for each neuron, yielding the quantity, Δ*F*/*F* (σ). Noise correlations were calculated as the correlation coefficient between mean-subtracted neural responses to tones, for each pair of neurons in an experiment.

### Statistical Analysis

To determine if values were different between experimental conditions to a statistically significant degree, we used a bootstrap hypothesis test (26). Given two datasets, *A* and *B*, having sample sizes of *n* and *m*, respectively, we tested *A* and *B* against the null hypothesis that they were drawn from a common distribution. The hypothesis test began by taking the absolute value of the observed difference of means, Δµ, between *A* and *B*. Next, we created the null distribution by pooling the individual values of *A* and *B*. Two sample sets, A* and B*, of size min(*n*,*m*), were randomly selected (with replacement) from the null distribution. The test statistic, Δµ*, was computed from the absolute value of the difference of the means obtained from the A* and B* sample sets. We repeated the random selection of A* and B* from the null distribution and the calculation of Δµ*, 10,000 times, to form a bootstrap distribution of Δµ*. *A* was taken to have a statistically significant different mean than *B*, if Δµ* was greater than Δµ in less than 5%, 1%, or 0.01% of the 10,000 bootstrapped values. This would mean that the probability was <5%, 1%, or 0.01% that samples in *A* and *B* came from a common distribution. All mean values are reported with standard errors of the mean (SEMs).

## RESULTS

### Psilocybin Has a Biphasic Effect on Auditory Cortical Responses to Sound

Figure 1*A* illustrates the timeline of our imaging experiments in awake mice. For each mouse, we began with a preinjection imaging session (Pre). We then waited an hour before an intraperitoneal (ip) injection of either a 2 mg/kg dose of psilocybin (14, 27, 28) dissolved in 0.1 mL saline (*n* = 6 mice, 2 females, 4 males) or the 0.1 mL saline vehicle alone (*n* = 3 mice, 3 males). Two of six psilocybin-treated mice received a dose on two separate experiments done 8 days apart, well beyond the ∼30-min half-life of psilocybin/psilocin in mice (23, 24). Each saline control mouse was imaged twice within several days. Psilocybin was provided by the NIDA Drug Supply Program.

The injected mouse was placed back under the microscope for postinjection imaging (Post 1), which typically began 5–8 min after injection. With the injected mouse still under the microscope, we then performed a final imaging session (Post 2), which typically began 34–43 min after injection. Each imaging session occurred in a different 2P field of view and lasted approximately 20 min while we presented 70 dB SPL, 0.5-s pure-tones at 10 frequencies (2–45 kHz) from a free-field speaker at randomized intertrial intervals of 6, 7, or 8 s. Bootstrap *t* test statistical comparisons (see materials and methods) were done between the pooled neuronal populations across experiments for each condition (i.e., Pre, Post 1, and Post 2 for both Saline and Psilocybin groups).

Figure 1*B*, *top*, shows the average neuronal response traces for Pre, Post 1, and Post 2 imaging sessions. Mean fluorescence values (Δ*F*/*F*) were normalized by the standard deviation of response amplitude (σ) for each neuron, yielding the quantity, Δ*F*/*F* (σ). Most neurons began each trial in a quiescent state for 2 s, followed by a sudden rise in activity after stimulus presentation (indicated by the horizontal black bars). To quantify neural response amplitudes, we averaged the activity during the 1 s following stimulus presentation for each neuron (Fig. 1*C*). Auditory responses were similar across Pre, Post 1, and Post 2 imaging sessions for mice injected with saline (bootstrap *t* test *P* > 0.05 for all comparisons). In contrast, psilocybin induced a biphasic modulation of neural responses to sound. Immediately after injection during psilocybin Post 1, neurons became hyper-responsive to sound, relative to the psilocybin Pre session (Pre vs. Post 1: bootstrap *t* test, *P* = 0.015). In contrast, 30-min postinjection during psilocybin Post 2, the average neural response amplitude decreased relative to psilocybin Post 1 (Post 1 vs. Post 2: bootstrap *t* test, *P* = 0.03).

### Psilocybin Increases Functional Connectivity in Auditory Cortex

It is important to note that the changes in neural response amplitudes after psilocybin injection occurred even though the acoustic stimulation remained unchanged, suggesting a change in internal brain state between Pre and Post 2 epochs. To investigate, we computed trial-to-trial correlations in response variance between pairs of neurons (25, 29, 30), i.e., “noise correlations.” An increase in noise correlation suggests an increase in functional connectivity between pairs of neurons (25, 29, 30). The average noise correlation for each experiment was computed, forming individual samples within a population that were then statistically compared using a nonparametric bootstrap *t* test (see materials and methods).

We measured noise correlations during Pre, Post 1, and Post 2 imaging sessions for both saline and psilocybin treatments (Fig. 1*D*, saline: *top*, psilocybin: *bottom*). We found that noise correlations tended to remain constant during Pre, Post 1, and Post 2 of saline treatments (bootstrap *t* test, *P* > 0.05 for all comparisons). In contrast, noise correlations significantly increased during psilocybin Post 2, compared with Pre (bootstrap *t* test, *P* = 0.044), indicating that psilocybin increased functional connectivity in A1 L2/3. Thus, our results suggest that psilocybin enhances the role of intrinsic brain control of sensory processing in A1 L2/3.

### Psilocybin Does Not Change Frequency Tuning in Auditory Cortex

**Figure 2.**
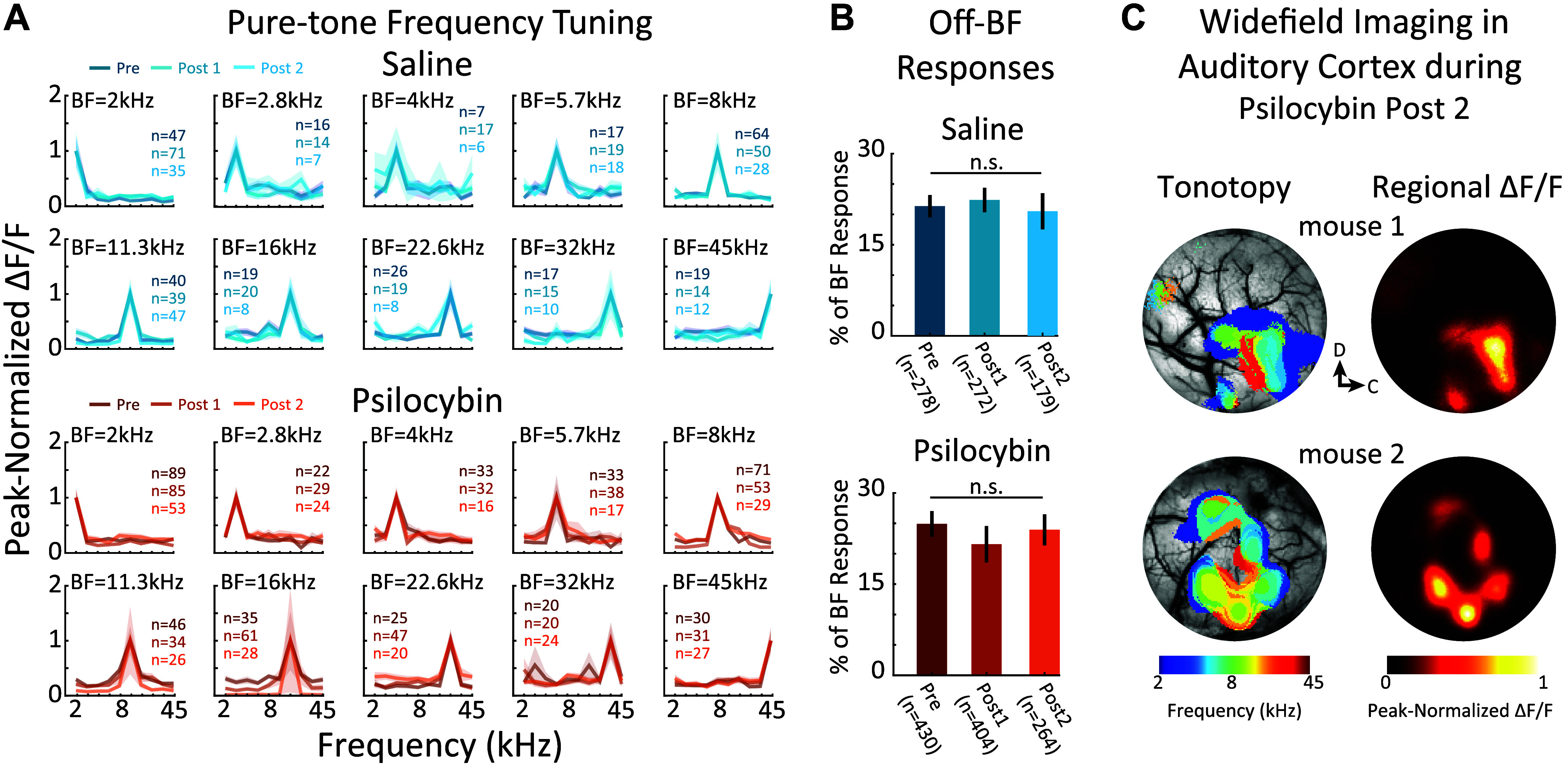
Psilocybin does not change frequency tuning in auditory cortex. *A*: frequency tuning curves (FTCs). FTCs from neurons with the same best frequency (BF) were averaged. Each panel contains three overlapping FTCs from the Pre, Post 1, and Post 2 conditions. Shading shows 1 SEM. *B*: average neural responses to off-BF frequencies. FTC selectivity was quantified as the average response to pure-tones off-BF (i.e., greater than or less than the BF) vs. at BF, for each neuron. Responses are reported as the percent of the response at BF. No change in FTC selectivity was found across conditions (bootstrap *t* test, n.s., *P* > 0.05 for all comparisons). *C*: tonotopy during psilocybin Post 2. Two mice were widefield imaged during psilocybin Post 2. The left column shows the tonotopic maps for each mouse. The right column shows the average regional fluorescence underlying the tonotopic maps for each mouse. Created with BioRender and published with permission.

So far, we have shown that psilocybin alters neuronal responses to sound in A1 L2/3. However, some aspects of auditory responsiveness in A1, such as pure-tone frequency selectivity, i.e., “frequency tuning,” arise from a frequency-specific labeled-line that originates in the cochlea. Thus, neuronal frequency tuning curves (FTCs) might persist despite the response amplitude changes induced by psilocybin. To investigate, we measured the FTC of each imaged neuron, and then grouped neurons by “Best Frequency’”(BF), i.e., the frequency corresponding to the FTC peak. We then peak-normalized each neuron’s FTC and averaged the FTCs for each BF. This produced 10 FTCs per experimental condition (Fig. 2*A*; *top row*: saline; *bottom row*: psilocybin). We found that FTCs were selective for the BF and were similarly shaped across all experimental conditions. We quantified changes in FTC shape by measuring the size of off-BF responses relative to BF (Fig. 2*B*). We found that the off-BF response magnitudes remained stable throughout experiments (bootstrap *t* test, *P* > 0.05 for all comparisons). Thus, psilocybin did not change the frequency tuning of individual neurons in A1 L2/3.

On the larger spatial scale of millimeters in auditory cortex, neuronal populations are organized across cortical space in pure-tone frequency gradients, i.e., in tonotopic maps (19, 25, 31). Since psilocybin did not seem to affect the FTCs of individual neurons, we used widefield Ca^2+^ imaging in two mice during psilocybin Post 2 to measure tonotopic maps. As expected, both mice showed clear tonotopic maps during psilocybin Post 2 (Fig. 2*C*). Our results indicate that while psilocybin modulates the amplitude and functional connectivity of neural responses, pure-tone frequency selectivity remains stable on both local and global scales within auditory cortex.

### Post 2 Effects Occur during a Psilocybin-Induced Hypoactive Behavioral State in Free-Roaming Mice

**Figure 3.**
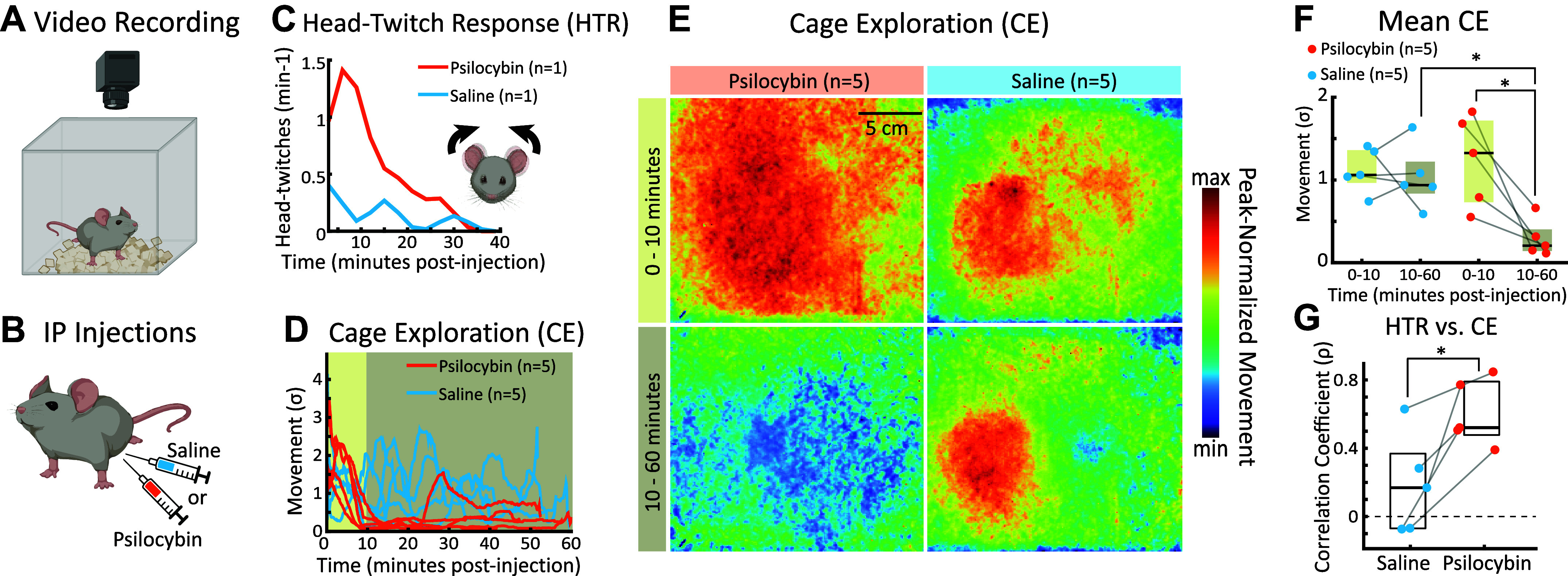
Psilocybin causes behavioral hypoactivity. *A*: video recording of behavioral activity. Injected mice were placed inside a video recording arena and their behavior was recorded for 40–60 min. *B*: experimental preparation. Mice were given an intraperitoneal injection of either saline or 2 mg/kg psilocybin. *C*: head-twitch response (HTR). Head-twitches were manually scored in two mice (*n* = 1 psilocybin; *n* = 1 saline). *D*: time-course of cage exploration (CE). The magnitude of mouse movement was quantified on a frame-by-frame basis and plotted for each treatment. *E*: CE across space. The magnitude of mouse movement across space in the video arena was averaged across mice for the psilocybin (*left*) and saline (*right*) groups. Movement was averaged in the 0–10 and 10–60 min time-windows. Hot and cool colors indicate regions with a high and low magnitude movement, respectively. The data in each heatmap were normalized to the peak movement value across heatmaps. *F*: mean CE. Movement was averaged in the 0–10 and 10–60 min time-windows for each mouse. The black horizontal bar and the rectangles show the median and interquartile range, respectively. The stars indicate significant differences (Saline 10–60 vs. Psilocybin 10–60: *, bootstrap *t* test, *P* = 0.011; Psilocybin 0–10 vs. 10–60: *, bootstrap *t* test, *P* = 0.012). *G*: similarity of HTR and CE. The CE time-course for each mouse was correlated against the HTR time-course. The psilocybin CE was more predictive of HTRs than the saline group (saline vs. psilocybin: *, bootstrap *t* test, *P* = 0.026). Created with BioRender and published with permission.

Having established a time-course for psilocybin’s effect on auditory cortex, we hypothesized that a similar time-course in the modulation of behavior may occur. To quantify psilocybin’s effects on the behavior of mice, we built a custom arena for video tracking of mouse movement (see materials and methods; Fig. 3*A*) after administering an intraperitoneal injection of either psilocybin (2 mg/kg in 0.1 mL saline) (*n* = 5 mice) or 0.1 mL saline vehicle (*n* = 5 mice) (Fig. 3*B*).

Previous research on the behavioral effects of psilocybin measured head-twitch responses (HTRs) as a dose-dependent indicator of psychedelic drug effects (28, 32) where the rate of head-twitches increased quickly after psilocybin injection, and then declined within 10 min. Here, we replicated previous findings of the HTR time-course by manually labeling video for each occurrence of a HTR, after both psilocybin (*n* = 1 mouse) and saline injections (*n* = 1 mouse; Fig. 3*C*, Supplemental Video S1). Consistent with previous work (28, 32), we found that the rate of head-twitches was greater for psilocybin than saline, increased quickly after psilocybin injection, and then declined within 10 min.

During behavioral labeling, we noticed that once HTRs declined, the mouse injected with psilocybin became very still within the clear box. To look more closely, we compared overall frame-by-frame behavioral movement across each video (i.e., cage exploration, CE) for mice injected with psilocybin versus saline (Fig. 3, *D* and *E*; see materials and methods for details; Supplemental Video S2 for HTRs observed in our experiments). The data show clear time-dependent differences between the movements of psilocybin versus saline mice: whereas saline mice tended to have a consistent amount of movement across the video session, psilocybin mice explored the arena during the first 10 min following injection, but then quickly became hypoactive and typically remained hypoactive for the rest of the session.

To quantify the extent of hypoactivity in each mouse, we averaged the movement during the first 10 min after psilocybin treatment (0–10 min in Fig. 3*D*; tan) and during the remainder of the video (10–60 min in Fig. 3*D*; brown). These populations were then statistically compared using a nonparametric bootstrap *t* test (see materials and methods). Figure 3*F* shows that there was no significant difference between the 0–10 versus 10–60 movement for saline mice (bootstrap *t* test, *P* = 0.66). In contrast, the psilocybin 10–60 movement was significantly less than both the psilocybin 0–10 movement (bootstrap *t* test, *P* = 0.012) and the saline 10–60 movement (bootstrap *t* test, *P* = 0.011).

It is important to note the similarity in the time-courses of the HTR and CE, which we quantified by taking the correlation coefficient between the CE trace for each mouse (Fig. 3*D*) and its corresponding HTR time-course “template” for saline and psilocybin conditions (Fig. 3*C*). The star in Fig. 3*G* shows that the average correlation coefficient was significantly greater for the psilocybin group (bootstrap *t* test, *P* = 0.026). Thus, both CE and HTRs show a similar time course in which behavioral activity declines ∼10 min after psilocybin injection. Our results show how a 2 mg/kg dose of psilocybin causes a prolonged period of hypoactivity in mice, lasting at least 60 min, and thus includes the time-window of our Post 2 imaging session in auditory cortex.

## DISCUSSION

Our results show that shortly after treating mice with a 2 mg/kg dose of psilocybin, there is an initial increase in neural responses to sound in A1 L2/3, but no change in the magnitude of behavioral activity. During the next 30–60 min after psilocybin treatment, cage exploration ceases and neural response amplitudes decrease, whereas pairwise noise correlations increase. Our results suggest that local functional connectivity in A1 L2/3 increased during the phase of behaviorally quiescent psychedelic effects.

We measured behavioral activity using a custom video arena and frame-difference analysis. Our motivation to capture gross movement came from noticing the wide range of behaviors that occurred after psilocybin injection. An alternative approach would have been to use supervised pose estimation software such as DeepLabCut (33) and SLEAP (34), or unsupervised methods of uncovering behavioral motifs such as VAME (35) and MoSeq (36). Although our analysis is fully automated and does not require data labeling or model training, future studies aiming to quantify how psilocybin affects specific mouse behaviors will benefit from pose estimation software.

Few studies have examined the response properties of individual neurons in awake mice during psilocybin treatment. Recently, it was shown that a 2 mg/kg dose of psilocybin in mice increased spiking and desynchronized local neural activity in the ACC ∼20 min after injection (14), and increased bold oxygen level-dependent responding and functional connectivity in numerous frontal, parietal, and temporal lobe areas, as well as the striatum (27). Although neither study probed A1 directly, nor examined sensory processing in general, the reported psilocybin-induced modulation of local cortical synchrony mimics the changes we find here in A1.

Our examination of the response properties of neurons in A1 revealed a transient increase in neural activity that preceded a subsequent increase in functional connectivity. These results indicate a modulation of stimulus- versus intrinsically driven influences in A1 L2/3, possibly reflecting a change in the levels of top-down versus bottom-up control in A1, which has been proposed as a mechanism by which the serotonergic psychedelic, lysergic acid diethylamide (LSD) affects activity in auditory and visual cortex (11, 18, 37). Previous work has shown that the orbitofrontal cortex (OFC) and ACC modulate auditory processing in A1 (38–41), and that 5-HT2a receptors are present in both rodent and human auditory cortex (42, 43), although one study suggests that the ability of a 5-HT2a agonist to induce hallucinogenic effects may rely on the additional activation of G(i/o) proteins (44). Thus, our observed changes in A1 might arise due to concurrent effects of psilocybin on activity and synchrony in frontal regions like OFC and ACC, which may change top-down control over A1 in a way that contributes to auditory alterations or pseudo hallucinations.

It is important to note that the fundamental auditory property of frequency tuning remained stable in our experiments despite the other dynamic effects of psilocybin, which speaks to the limits in which hearing can be altered during a psychedelic experience. Our findings are strikingly similar to a previous study on the effects of the serotonergic psychedelic, 2,5-dimethoxy-4-iodoamphetamine (DOI), on primary visual cortex in awake mice (45): the binding of DOI to 5-HT2a receptors is thought to reduce visual response amplitudes while leaving stimulus selectivity intact. Therefore, the changes in perception across various senses during serotonergic psychedelic experiences may stem from similar modulations in neural responsiveness within distinct sensory regions of the cortex.

Importantly, both the stability of tuning and the increase in functional connectivity that we observed during the later behaviorally quiescent phase of psilocybin argue against an arousal-based brain-state change. Previous work focusing on arousal and auditory perception more generally suggest that decreases in arousal predict changes in tuning (46–48), and this pattern is largely absent in our results. Although we do see decreases in neural responsivity in the Post 2 time-period, we do not see the anticipated changes in neural selectivity to pure-tones. Thus, our findings more likely reflect the effects of serotonergic psychedelics binding to 5-HT2a, and perhaps also 5-HT1a receptors in auditory cortex (42, 43, 49).

The link between psilocybin and the psychological state of arousal is complex, and presently understudied. Recent studies have suggested that high levels of arousal are linked to locus coeruleus activation, increased norepinephrine levels and pupil dilation (50). Although psilocybin can alter pupil dilation, its correlation with arousal is unclear, and, at least one case study reports that chronic use of multiple psychedelics can produce permanent pupil dilation (51). The impact of psilocybin on other neuromodulatory systems such as acetylcholine or norepinephrine is open for future research. One study showed that psilocybin reduces norepinephrine levels in the hypothalamus of rats (52), and a recent study in humans suggests that in patients being treated with a selective norepinephrine and serotonin reuptake inhibitors (SNRIs and SSRIs, respectively), the effect of psilocybin on depressive symptoms is reduced when compared with non-SNRI/SSRI treated individuals who are given the same dose of psilocybin (53). Collectively, these findings highlight that psilocybin interacts with neuromodulatory systems that support arousal in novel ways, and that future research is needed to better identify the relevant mechanisms.

In summary, our findings speak to the role of psilocybin in modulating the neural basis of sensory perception. Although psilocybin shows great promise as a therapeutic adjuvant, it remains unclear how psilocybin produces its therapeutic effects. Our findings outline how psilocybin affects the activity of individual neurons in an awake mammal, paving the way for further in vivo investigation of psilocybin’s effects on cortical microcircuits.

## DATA AVAILABILITY

Data will be made available upon reasonable request.

## SUPPLEMENTAL DATA

10.5281/zenodo.11069505 Supplemental Videos S1 and S2: https://zenodo.org/doi/10.5281/zenodo.11069505.

## GRANTS

This research was funded by the University of Maryland (UMD) Individual Grand Challenges Award GC30 (to Adam T. Brockett), and a UMD Brain and Behavior Institute seed grant (to Nikolas A. Francis).

## DISCLOSURES

No conflicts of interest, financial or otherwise, are declared by the authors.

## AUTHOR CONTRIBUTIONS

A.T.B. and N.A.F. conceived and designed research; N.A.F. performed experiments; N.A.F. analyzed data; A.T.B. and N.A.F. interpreted results of experiments; N.A.F. prepared figures; A.T.B. and N.A.F. drafted manuscript; A.T.B. and N.A.F. edited and revised manuscript; A.T.B. and N.A.F. approved final version of manuscript.
